# Resolvin D2 protects against cerebral ischemia/reperfusion injury in rats

**DOI:** 10.1186/s13041-018-0351-1

**Published:** 2018-02-13

**Authors:** Gang Zuo, Dongping Zhang, Rutao Mu, Haitao Shen, Xiang Li, Zhong Wang, Haiying Li, Gang Chen

**Affiliations:** Department of Neurosurgery & Brain and Nerve Research Laboratory, The First Affiliated Hospital of Soochow University, 188 Shizi Street, Suzhou, 215006 China

**Keywords:** Ischemic stroke, Cerebral ischemia/reperfusion injury, Resolvin D2, ω-3 fatty acid, GPR18, Neuron, Brain microvascular endothelial cell

## Abstract

Cerebral ischemia/reperfusion (I/R) injury is a critical factor leading to a poor prognosis for ischemic stroke patients. ω-3 fatty acid supplements taken as part of a daily diet have been shown to improve the prognosis of patients with ischemic stroke. In this study, we aimed to investigate the potential effects of resolvin D2 (RvD2), a derivative of ω-3 fatty acids, and its possible advantage on cerebral I/R injury in rats. Cerebral I/R caused by middle cerebral artery occlusion and reperfusion (MCAO/R) was established in Sprague-Dawley rats. First, in rats fed a regular diet, the MCAO/R stimulus led to a significant decrease in endogenous production of RvD2. Exogenous supply of RvD2 via intraperitoneal injection reversed MCAO/R-induced brain injury, including infarction, inflammatory response, brain edema, and neurological dysfunction. Meanwhile, RvD2 reversed the MCAO/R-induced decrease in the protein level of GPR18, which has been identified as a receptor for RvD2, especially in neurons and brain microvascular endothelial cells (BMVECs). Furthermore, RvD2 exerted rescue effects on MCAO/R-induced neuron and BMVEC death. Moreover, GPR18 antagonist O-1918 could block the rescue effects of RvD2, possibly at least partially though the GPR18-ERK1/2-NOS signaling pathway. Finally, compared with ω-3 fatty acid supplements, RvD2 treatment had a better rescue effect on cerebral infarction, which may be due to the MCAO/R-induced decrease in 5-lipoxygense phosphorylation and subsequent RvD2 generation. In conclusion, compared with ω-3 fatty acids, RvD2 may be an optimal alternative and complementary treatment for ischemic stroke patients with recanalization treatment.

## Introduction

Ischemic stroke is a common cause of long-term disability and is the second leading cause of death worldwide. Recanalization therapy provokes cerebral ischemia/reperfusion (I/R) injury, including vascular permeability increase, blood-brain barrier (BBB) disruption, and brain edema [1, 2]. Brain microvascular endothelial cells (BMVECs), the main cells composing the BBB, are connected by tight junction proteins such as zonula occludens 1 (ZO-1) [3]. BMVECs represent main targets for the reactive oxygen species (ROS) and inflammatory response produced during cerebral I/R [4]. Neuronal apoptosis and necrosis are other central pathological processes in I/R-induced brain injury. Inflammatory cytokines such as tumor necrosis factor α (TNF-α) and interleukin 6 (IL-6), which are stimulated after necrosis induction, significantly increase in ischemic and reperfusion-induced brain tissue inflammation [5]. Therefore, the protection of BMVECs and neurons against I/R-induced oxidative stress and inflammatory response may be a potential therapeutic target for ischemic stroke patients with recanalization treatment [4].

Resolvin D2 (RvD2), a member of the resolvin family, is produced from ω-3 polyunsaturated fatty acids after a series of catalyzed reactions by lipoxygenases [6]. Notably, ω-3 fatty acids are able to cross the BBB effectively via its receptors to enter brain tissue [7]. It has been shown that both RvD2 and resolvin D1 can regulate the chronic inflammatory process that occurs in the adipose tissue of obese patients [8]. Another study found a direct correlation between the cerebrospinal fluid (CSF) levels of RvD2 in the post-mortem brain and the cognitive scores of patients with Alzheimer’s disease [9]. In addition, aging rats treated with DHA were found to exhibit improved memory, which was accompanied by increased levels of RvD2 in the brain [9, 10]. Moreover, the anti-inflammatory action of RvD2 is dependent on its G protein-coupled receptor (GPCR), GPR18, the mechanisms of which have not yet been elucidated [11, 12].

Advanced research has reported that fish oil taken as a supplement apart from daily diet may improve the clinical outcomes of patients with ischemic stroke. One study indicated that oral ω-3 fatty acids could reduce cerebral infarction and the frequency of symptomatic vasospasm, and could also improve clinical prognosis [13]. In addition, our previous study found that ω-3 fatty acids could protect early brain injury (EBI) from subarachnoid hemorrhage (SAH) [14]. The human body converts ω-3 fatty acids into RvD2 through the lipoxygenase (LOX) metabolism: 12/15-lox can promote DHA conversion to 17-HDHA, and 17-HDHA then generates 17-HPDHA, which can be metabolized to RvD2 in a 5-LOX phosphorylation-dependent manner [15].

However, no study has yet reported the effects of RvD2 on I/R-induced brain injury. Therefore, the aim of this study was to investigate the effects of RvD2 on I/R-induced brain injury in a rat MCAO/R model as well as its underlying mechanisms.

## Results

### MCAO/R led to a significant decrease in the endogenous production of RvD2

**Fig. 1:**
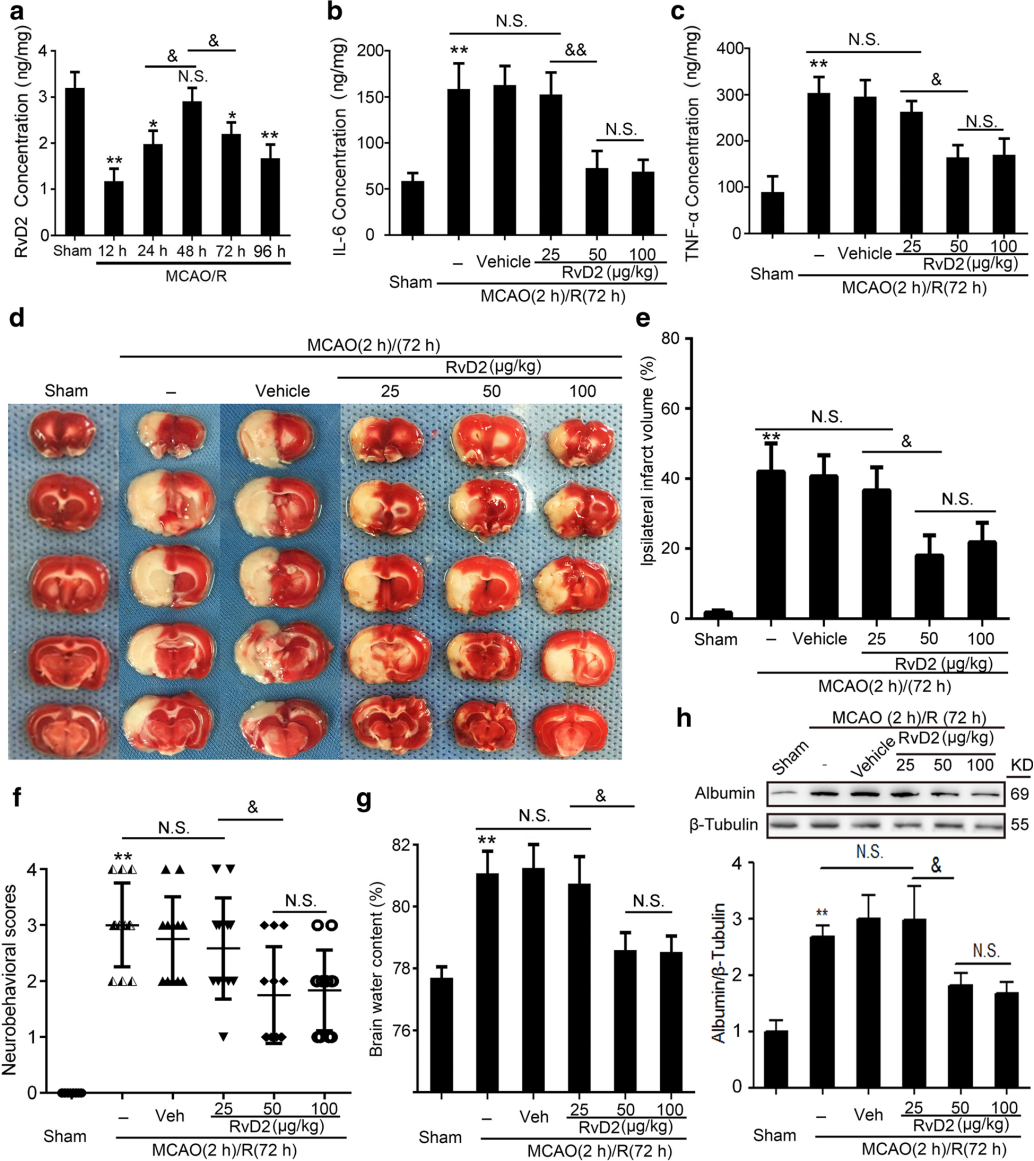
Endogenous production of RvD2 and effects of exogenous RvD2 treatment on brain I/R injury. **a** The level of the endogenous RvD2 after MCAO/R in brain tissue was detected by ELISA at 12, 24, 48, 72, and 96 h. **b** and **c** IL-6 and TNF-α secretions were determined by ELISA after different doses of RvD2 treatment for MCAO/R in brain tissue. **d** TTC-stained coronal sections from representative animals at 72 h after reperfusion. The infarct volumes and reproducible infarct volumes were observed. **e** The infarct volume was expressed as a percentage of the ipsilateral hemispheric volume. **f** The neurobehavioral score was shown as the median, with interquartile range. **g** Brain water content was detected. **h** The protein levels of albumin were detected by Western blot, and the mean value of the sham group was normalized to 1.0. In (**a**–**c**, **e**–**h**), data are means ± SEM. *P < 0.05, **P < 0.01 vs. sham group; ^&^*P* < 0.05, ^&&^*P* < 0.01, N.S. = no significant differences, *n* = 6

In order to elucidate the endogenous production of RvD2 after I/R, we detected the RvD2 level in brain tissue after MCAO/R by enzyme-linked immunosorbent assay (ELISA) in rats fed a regular diet. The results revealed that the endogenous RvD2 levels were significantly decreased in the MCAO/R group compared with the sham group (Fig. 1a). At MCAO (2 h)/R (48 h), the levels of RvD2 had a restorative effect.

### Exogenous supply of RvD2 reversed MCAO/R-induced brain injury

Next, we used exogenous RvD2 (25, 50, and 100 μg/kg) to treat the MCAO/R rats and detected its effects on inflammatory cytokine production and brain injury after MCAO/R. We found that the levels of IL-6 and TNF-α in infarct-side brain tissue were significantly increased after MCAO/R (72 h), but were significantly decreased by 50 μg/kg and 100 μg/kg RvD2 treatment (Fig. 1b and c). Triphenyltetrazolium chloride (TTC) staining showed that the infarct area significantly decreased with 50 and 100 μg/kg RvD2 treatment (Fig. 1d and e). Compared with the sham group, the neurological score of the MCAO/R group was significantly higher, suggesting a remarkable neurological defect induction by the I/R model. However, the neurological scores of the MCAO/R + RvD2 (50 and 100 μg/kg) groups were significantly lower than that of the MCAO/R group (Fig. 1f). Additionally, brain water content was found to be significantly lower in infarct-side brain tissue samples of the RvD2 treatment group than in vehicle group rats, suggesting that RvD2 treatment effectively rescues the MCAO/R-induced BBB leakage (Fig. 1g). These findings were further confirmed by Western blot assays of brain albumin content (Fig. 1h). These results were consistent with the effects of RvD2 on inflammatory cytokine production.

### RvD2 reversed the MCAO/R-induced decrease in the protein level of GPR18, especially in neurons and BMVECs

**Fig. 2:**
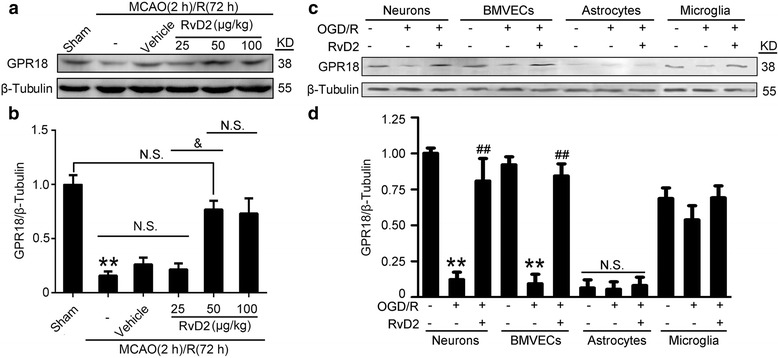
RvD2 reversed the MCAO/R-induced decrease in the protein level of GPR18. **a** Western blot analysis was used to measure the protein levels of GPR18 after I/R brain injury. **b** The GPR18 expression levels were quantified. Data are presented as mean ± SEM, *n* = 6. **P < 0.01 vs. sham group, ^&^*P <* 0.05. N.S. = no significant differences. **c** Western blot analysis was used to measure the protein levels of GPR18 in cultured neurons, astrocytes, microglia, and BMVECs under indicated treatments. **d** The GPR18 expression levels were quantified. The level of GPR18 in the normal neuron group was normalized to 1.0. Data are presented as mean ± SEM, *n* = 3. **P < 0.01 vs. normal group, ^##^*P <* 0.01. N.S. = no significant differences

To identify the mechanism underlying the neuroprotective effect of RvD2, we first examined the protein level of GPR18 which has been identified as a receptor for RvD2. Western blot analysis showed that the protein level of GPR18 was down-regulated in the MCAO/R group compared to the sham group, whereas 50 μg/kg RvD2 treatment could reverse the decrease of GPR18 (Fig. 2a and b). The changes in GPR18 were consistent with results of previous experiments [16]. To further distinguish the cell type specificity of GPR18 expression, a Western blot assay was performed on cultured neurons, microglia, astrocytes, and brain microvascular endothelial cells (BMVECs). The results showed that GPR18 was mainly expressed in neurons, microglia, and BMVECs under normal conditions, with very little expression in astrocytes under both normal and OGD/R conditions (Fig. 2c and d). Compared with the normal group, the protein levels of GPR18 in neurons and BMVECs showed about a 3.5-fold decrease at 72 h after OGD/R, while OGD/R induced only a slight decrease in the protein level of GPR18 in microglia (Fig. 2c and d). In addition, the OGD/R-induced decrease of GPR18 in neurons and BMVECs was reversed by RvD2 treatment, while RvD2 treatment did not induce a significant change in the protein level of GPR18 in microglia exposed to OGD/R (Fig. 2c and d). Based on these results, we focused on the roles of GPR18 in neurons and BMVECs in the following study.

### RvD2 exerted rescue effects on MCAO/R-induced neuron and BMVEC death

**Fig. 3:**
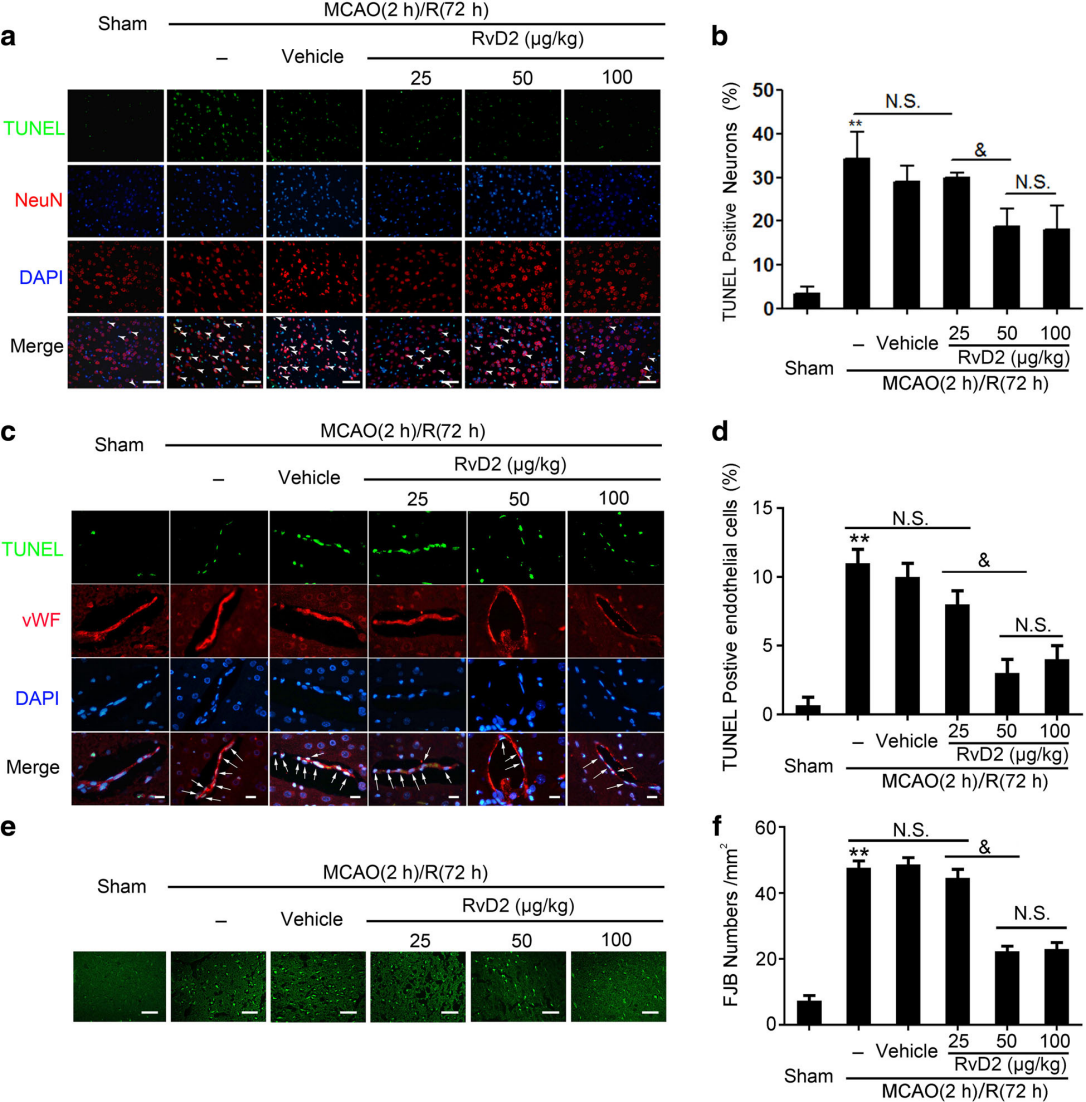
RvD2 exerted rescue effects on MCAO/R-induced neuron and BMVEC death. Apoptotic cell death was detected by terminal deoxynucleotidyl transferase dUTP nick end labeling (TUNEL) staining. Sections were labeled by TUNEL (green) to detect apoptotic neurons (red) (**a**) and brain microvascular endothelial cells (BMVECs) (red) (**c**) and counterstained with DAPI (blue) to detect nuclei. Arrows point to TUNEL-positive cells. The percentage of TUNEL-positive brain cells was calculated (**b** and **d**). Scale bar = 64 μm (A) and 24 μm (**c**). Data are expressed as mean ± SEM, *n* = 6. **P < 0.01 vs. sham group, ^&^*P* < 0.05, N.S. = no significant differences. **e** Fluoro-Jade B (FJB) staining was used to detect necrosis. Scale bar = 64 μm. The number of FJB-positive brain cells was calculated (**f**). Data are expressed as mean ± SEM, *n* = 6. **P < 0.01 vs. sham group, ^&^*P* < 0.05, N.S. = no significant differences

We further explored the effects of RvD2 (25, 50, and100 μg/kg) on cell death in the ischemic penumbra of brain tissues at 72 h after MCAO/R by TUNEL and FJB staining. Compared with the sham group, TUNEL-positive neurons were significantly increased in the MCAO/R group, while the numbers of TUNEL-positive neurons were reduced with RvD2 treatments (Fig. 3a and b). In addition, at 72 h after MCAO/R + RvD2 (50 μg/kg and 100 μg/kg), TUNEL-positive neurons were most obviously decreased compared with the MCAO/R+ RvD2 (25 μg/kg) group. Consistently, the apoptotic index of BMVECs showed the same trend (Fig. 3c and d). Meanwhile, FJB-positive cells in the MCAO/R group were significantly increased compared with the sham group, while the number of FJB-positive brain cells was dramatically reduced in the RvD2 (50 and 100 μg/kg) group at 72 h after I/R (Fig. 3e and f).

### The neuroprotective effects of RvD2 were dependent on its receptor GPR18

**Fig. 4:**
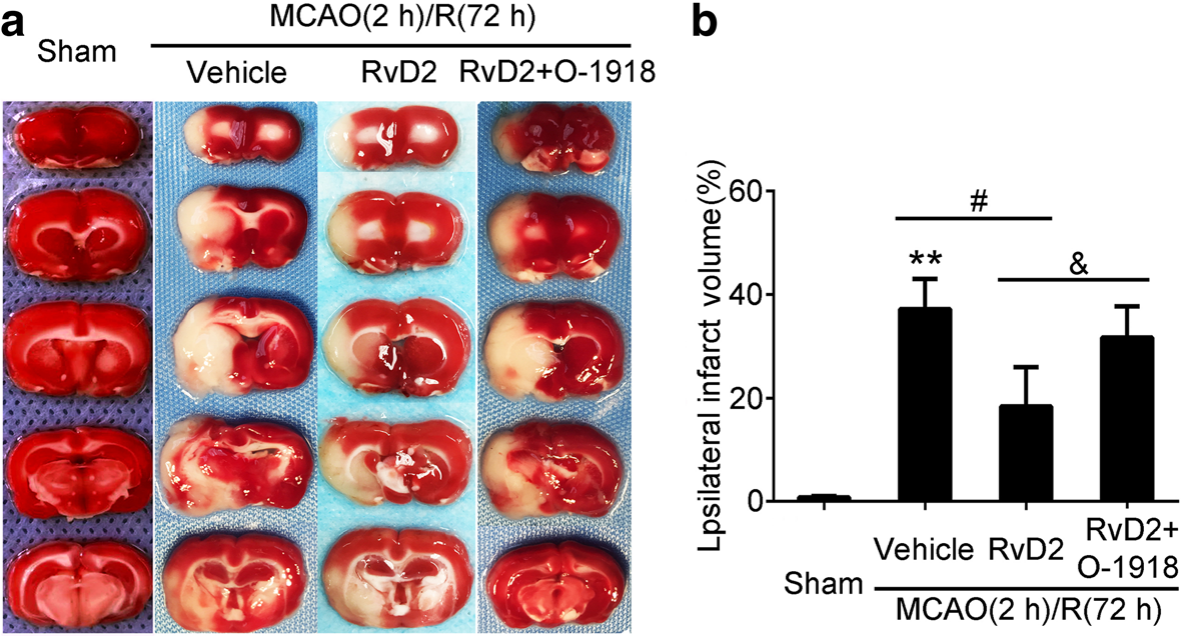
The rescue effects of RvD2 were reversed by GPR18 antagonist O-1918 after MCAO/R. **a** TTC-stained coronal sections from representative animals at 72 h after reperfusion. **b** Statistical analysis of infarct volumes. The infarct volume was expressed as a percentage of the ipsilateral hemispheric volume. Data were means ± SEM. *P < 0.05, **P < 0.01 vs. sham group, ^#^*P* < 0.05, ^&^*P <* 0.05, n = 6

Furthermore, we used O-1918, an antagonist of GPR18, to detect the mechanism underlying the neuroprotective function of RvD2. TTC staining showed that infarct volume was significantly increased in the RvD2 + O-1918 group compared to the RvD2 group under I/R conditions (Fig. 4a and b), which indicated that the neuroprotective effects of RvD2 were dependent on GPR18.

### RvD2 exhibited rescue effects through the GPR18-ERK1/2-NOS signaling pathway

**Fig. 5:**
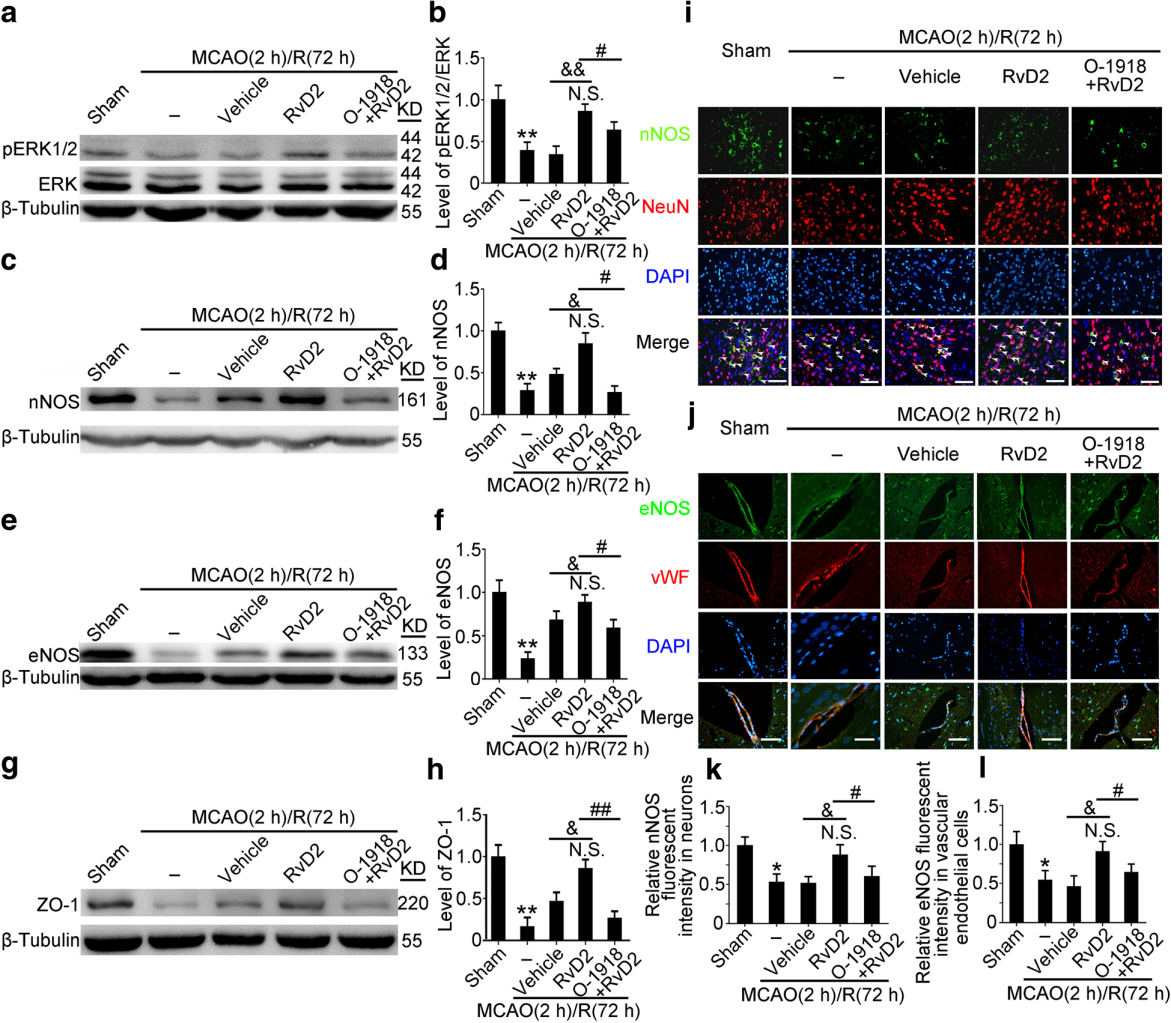
RvD2 promoted ERK/NOS pathway in GPR18-dependent manner after MCAO/R. **a**–**h** The protein levels of pERK 1/2, ERK1/2, ZO-1, nNOS, and eNOS were assessed by Western blot and quantified. The mean values of the protein levels of pERK1/2, ERK, ZO-1, nNOS, and eNOS in the sham group were normalized to 1.0. Data are means ± SEM, n = 6. **P < 0.01 vs. sham group, ^#^*P <* 0.05, ^##^*P* < 0.01, ^&^*P <* 0.05, ^&&^*P* < 0.01. N.S. = no significant differences. **i** and **j** Double immunofluorescence analysis was performed with nNOS antibodies or eNOS antibodies (*green*) and neuron marker (NeuN, *red*) or endothelial cell marker (vWF, *red*), and the nuclei were fluorescently labeled with 4,6-diamino-2-phenylindole (DAPI) (*blue*). Arrows point to nNOS-positive neurons or eNOS-positive endothelial cells. Scale bar = 32 μm. The relative fluorescent intensity of nNOS in neurons and eNOS in endothelial cells was quantified (**k** and **l**). The mean value of the intensity of fluorescence in the sham group was normalized to 1.0. Data are means ± SEM, n = 6. *P < 0.05 vs. sham group, ^#^*P <* 0.05, ^&^*P <* 0.05. N.S. = no significant differences

Next, we found that RvD2 promoted ERK1/2 phosphorylation, which was inhibited by O-1918 treatment (Fig. 5a and b). In addition, the protein levels of neuronal NOS (nNOS) and endothelial NOS (eNOS) were significantly abated in the ischemic hemisphere after MCAO/R (72 h). Meanwhile, the production of nNOS, eNOS, and ZO-1 were significantly enhanced after treatment with RvD2 at 72 h after reperfusion (Fig. 5c–l). And, the O-1918 treatment could partly block the function of RvD2 (Fig. 5).

### RvD2 exerted a better rescue effect on cerebral infarction than ω-3 fatty acids

**Fig. 6:**
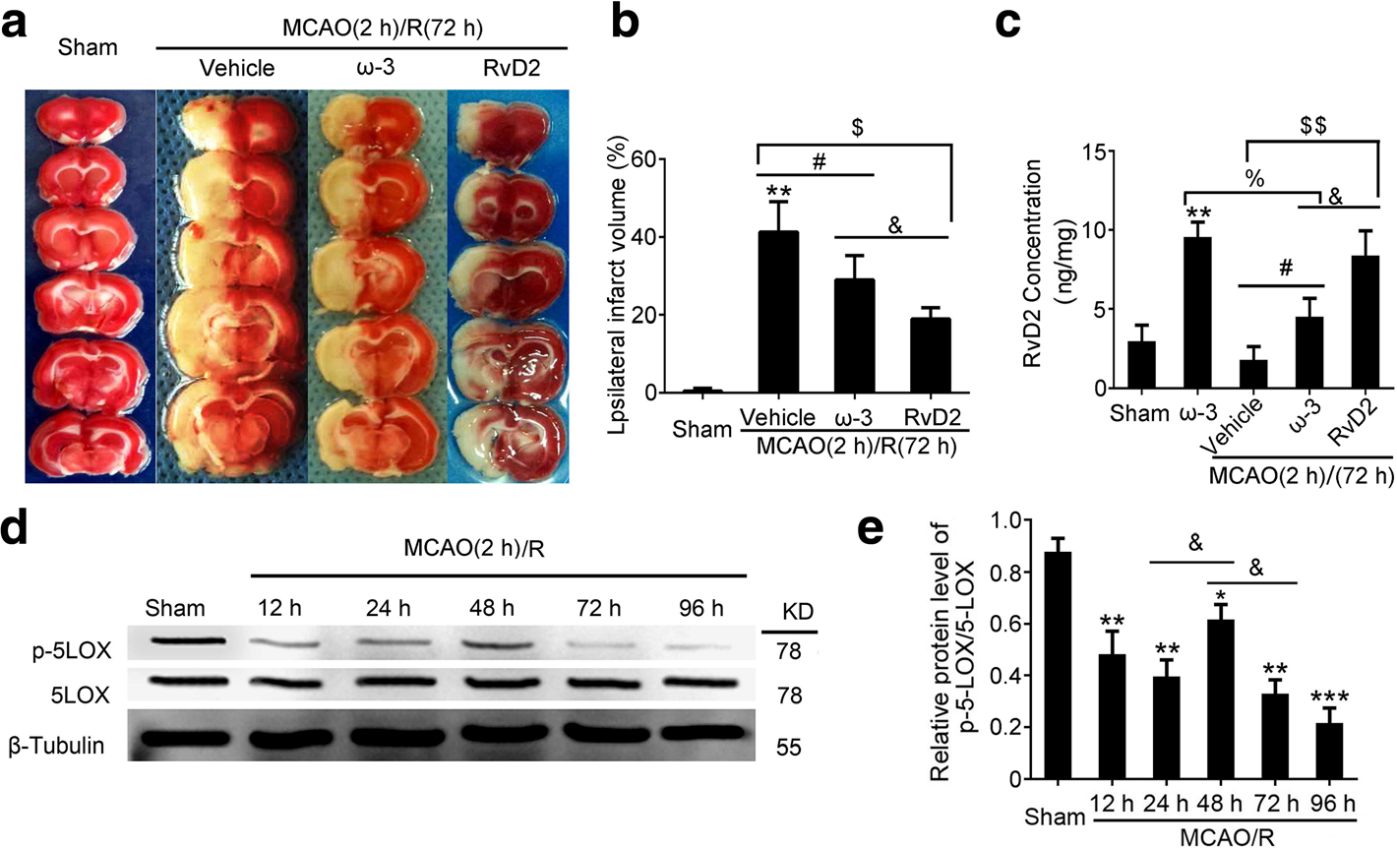
ω-3 fatty acids was partly disabled after MCAO/R. **a** TTC-stained coronal sections from representative animals at 72 h after reperfusion. **b** Statistical analysis of infarct volume. Reproducible infarct volumes were observed in the MCAO/R + vehicle group, MCAO/R + ω-3 group, and MCAO/R + RvD2 group. The infarct volume is expressed as a percentage of the ipsilateral hemispheric volume. Data are means ± SEM. **P < 0.01 vs. sham group, ^#^*P* < 0.05, ^$^*P* < 0.05, ^&^*P* < 0.05, n = 6. **c** RvD2 production after supplying ω-3 fatty acids and injection of RvD2 was detected by ELISA. Data are means ± SEM, n = 6. **P < 0.01 vs. sham group, ^#^*P* < 0.05, ^&^*P* < 0.05, ^%^*P* < 0.05, ^$$^*P* < 0.01. N.S. = no significant differences. **d** Western blot analysis was used to detect the protein levels of 5-LOX and Phos-5-LOX after I/R brain injury. Quantification of the Phos-5-LOX/5-LOX level is shown (**e**). Data are presented as mean ± SEM, n = 6. **P < 0.01, ***P < 0.001 vs. sham group, ^&^*P <* 0.05, N.S. = no significant differences

Because ω-3 fatty acids are precursors of RvD2, we also compared the different effects of injecting RvD2 directly and supplying ω3-polyunsaturated fatty acids. TTC staining was performed to observe the infarct area of the ischemic hemisphere, and we found that the volume of the ischemia was significantly decreased after the RvD2 or ω-3 treatment, while RvD2 seemed to be more effective at protecting I/R against infarction (Fig. 6a and b).

### MCAO/R induced a decrease in 5-LOX phosphorylation and subsequent RvD2 generation

In the sham group, supplementation of ω-3 fatty acids promoted RvD2 production, whereas the RvD2 synthesis was restrained after MCAO/R, indicating that the effects of ω-3 fatty acids were disabled after MCAO/R (Fig. 6c). Because LOX activity was involved in converting ω-3 into RvD2, we determined the phosphorylation of 5-LOX in the brain by Western blot. Phosphorylated 5-LOX in brain tissue was much lower than that in the sham group after MCAO/R (Fig. 6d and e), which was in accordance with the change of RvD2 production after MCAO/R as shown in Fig. 1a. These results indicated that MCAO/R-induced a decrease in 5-LOX phosphorylation and subsequent RvD2 generation.

## Discussion

Cerebral ischemia is one of the main causes of death around the world. The clinical treatment of cerebral ischemia is limited, and new neuroprotective therapies are desperately needed. In the present study, we found that the generation of endogenous RvD2 was decreased and the supplementation of exogenous RvD2 played a role in neuroprotection after MCAO/R. To date, information on the generation and function of RvD2 in the brain was very limited.

**Fig. 7:**
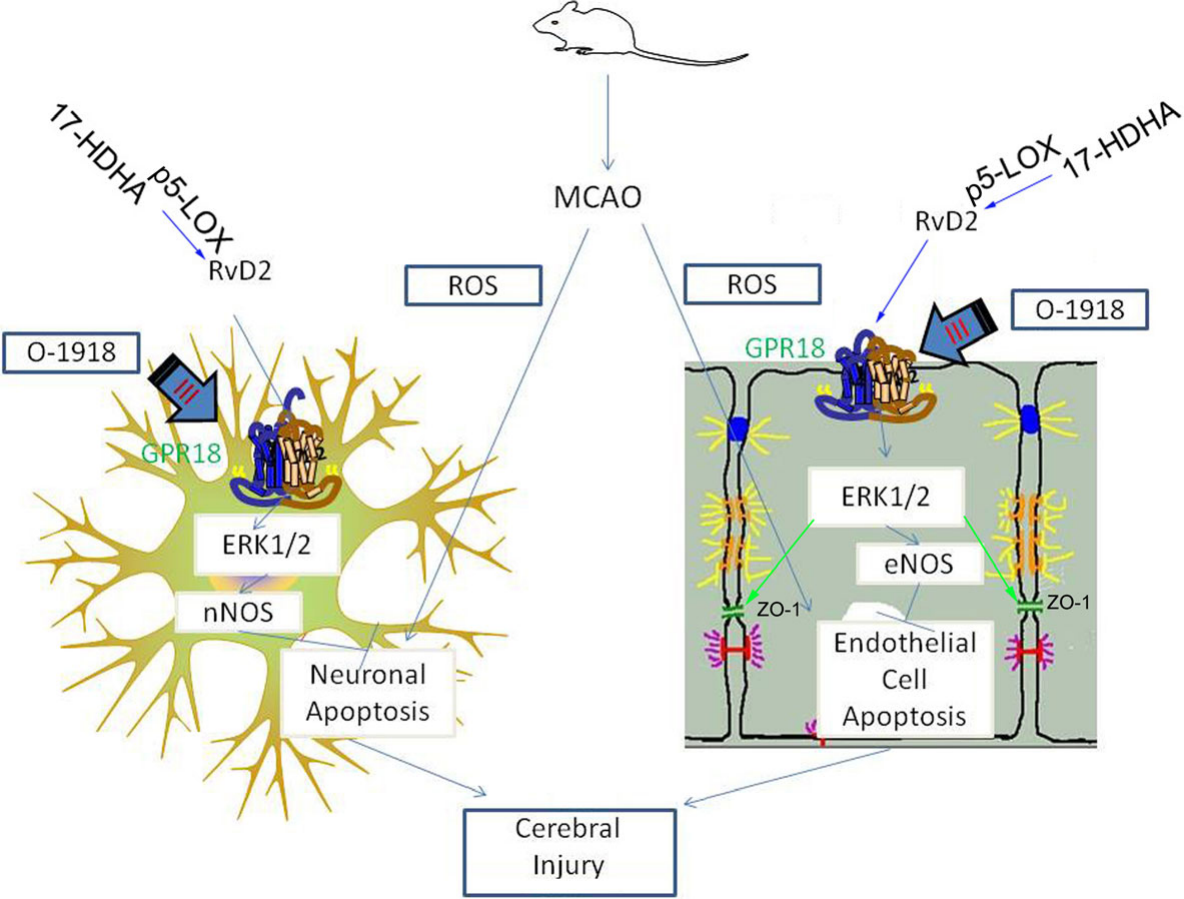
Mode pattern illustrating the possible mechanisms of RvD2’s alleviation of cerebral ischemic and reperfusion injury. The intracellular ROS in neurons and endothelial cells were active and induced cell apoptosis and cerebral injury after MCAO/R induction. Extracellular 17-HDHA generated RvD2 depending on the phosphorylation of 5-LOX. RvD2 bound to its receptor GPR18 in the plasma membrane and triggered intracellular signal transduction through phosphorylation of ERK1/2, followed by stimulating nNOS or eNOS to suppress neuronal or endothelial cells apoptosis and induced an increase of tight junction protein ZO-1 to maintain BBB integrity. This pathologic process alleviated early brain injury after MCAO/R

The existence of RvD2 was discovered in the brains of mice subjected to stroke in a study aimed to develop methods for the detection of resolvins, indicating that it could be involved in the improvement of ischemic lesions [17]. It was reported that RvD2 could reverse LPS-induced depression-like behaviors through the mTORC1 signaling pathway [18]. Consistently, our results confirmed that a cessation of the generation of resolvin in the brain could be related to the brain injury after ischemic stroke. Therefore, exogenous RvD2 was able to reduce the release of TNF-α and IL6 in the brain, decrease the infarct area, and protect the neurons and BMVECs cells from apoptosis and necrosis after MCAO/R (Fig. 7). Although previous studies reported the protective effects of RvD2 on brain regions [10, 19], to our knowledge, these are the first reported results to suggest that RvD2 acts on I/R brain injury.

Moreover, the possible mechanisms of the action of RvD2 have not been clarified. In 2015, Nan et al. presented evidence that GPR18 was expressed on human leukocytes as a specific cell surface GPCR for RvD2 [20]. GPRs, composed of seven transmembrane domains, are the largest family of receptors and regulate various physiological processes. One of these receptors, GPR18, consists of 331 amino acids and is conserved in mammals, which was originally described in the human spleen and testes [21]. Moreover, the expressions and distributions of GPR18 were evaluated in the hypothalamus neurons on diet induced obesity. Additionally, GPR18 was found to be extensively distributed in the central nervous system (CNS), including in microglia, and play a significant role in the regulation of microglia in the CNS [22]. Another study, in which RvD2 was injected into the spine to treat chronic pain, suggested that sensory neurons are responsive to this resolvin and may express GPR18 [23]. As expected, we found that the expression of GPR18 was inhibited after MCAO/R, which was reversed by RvD2 treatment. In addition, the neuroprotective effects of RvD2 were significantly abolished by O-1918, which was identified as an antagonist for GPR18 as a structurally related synthetic compound. Therefore, we confirmed that RvD2 exerts its effects through GPR18 to transfer the signaling in neurons and BMVEC cells. In our study, based on the cell type specificity of GPR18 expression shown in Fig. 2c and d, we focused only on the effects of RvD2 on neuron apoptosis and BBB maintenance. The mechanisms underlying the role of RvD2 in inflammation by GPR18 expression in microglia require further research.

It is known that ROS induces a series of oxidative stress reactions and leads to brain cell apoptosis and necrosis during I/R stroke onset. NO can be produced by three different isoforms of the enzyme NOS to play the role of an antioxidant. nNOS is expressed in specific neurons of the CNS and is involved in synaptic plasticity. Among the NOSs, eNOS, as the most important isoform, can keep blood vessels dilated and control blood pressure, which has numerous vasoprotective and anti-atherosclerotic functions [24]. In the present study, nNOS and eNOS, considered anti-inflammation biosynthesizers, were significantly enhanced after RvD2 post-treatment in the MCAO/R model. After activation of GPR18, the synthesis of NOS could be controlled by the ERK1/2 pathway [25]. ERK plays an essential role in many cellular and physiological processes, including cell proliferation, differentiation, survival, migration, invasion, transcription, metabolism, and apoptosis. The expression of a subset of tight junction (TJ) proteins such as ZO-1 also depends on ERK1/2 phosphorylation [26]. In our study, we found that the decreases in ERK1/2 phosphorylation and ZO-1 expression were significantly ameliorated by RvD2 treatment after MCAO/R, resulting in improved nNOS and eNOS levels, thereby maintaining BBB integrity.

In addition, RvD2 was metabolized by ω-3 fatty acids, especially DHA, which was separated from fish oil. In recent years, fish oil has been favored by both consumers and academics, and its brain protection in ischemic stroke has garnered a great deal of interest. Pretreatment with fish oil or its constituent ω-3 fatty acids has been shown to alleviate I/R-induced brain injury [27–29], suggesting that fish oil can effectively reduce I/R damage. However, post-treatment is a much more likely scenario in clinical treatment. Correia et al. found that DHA pretreatment could effectively improve the memory recovery function of cerebral ischemia rats, but DHA postconditioning did not have the same effect [30]. Additionally, Yang found that DHA post-treatment increased rat brain I/R injury [31]. Here, we found that the capacity of ω-3 fatty acids to generate RvD2 was reduced by MCAO/R, making them less effective than direct RvD2 injection, suggesting that in the early brain I/R injury process, the metabolic process of fish oil (especially DHA) may be blocked. Furthermore, LOX played a critical role in the conversion of DHA into RvD2: 12/15-lox could promote DHA conversion to 17-HDHA, and 17-HDHA then generated 17-HPDHA, which could be metabolized to RvD2 after 5-LOX phosphorylation. A recent study suggested that phosphorylation of 5-LOX at serine 663 could stimulate lipoxin production, which plays a role in the last steps of RvD2 synthesis [32]. In this study, we found that the level of p5-LOX was significantly decreased after MCAO/R, leading to the metabolic failure of ω-3 fatty acids.

This study demonstrated that the functions of ω-3 fatty acids were partly blocked by the decrease in p5-LOX after I/R injury. For the first time, RvD2 was reported to alleviate cerebral I/R-induced brain injury, suggesting that it could be a novel neuroprotective candidate.

## Methods

### Animal and ethics approval and consent to participate

Adult male Sprague-Dawley (SD) rats weighing 250–300 g were provided by the Animal Center of the Chinese Academy of Sciences, Shanghai, China. The rats were housed in animal quarters under controlled temperature and humidity with a 12 h light/dark cycle. All animal procedures are strictly in accordance with the guideline of the Ethics Committee of Soochow University on animal care and use.

### Rat model of cerebral I/R and groups

**Fig. 8:**
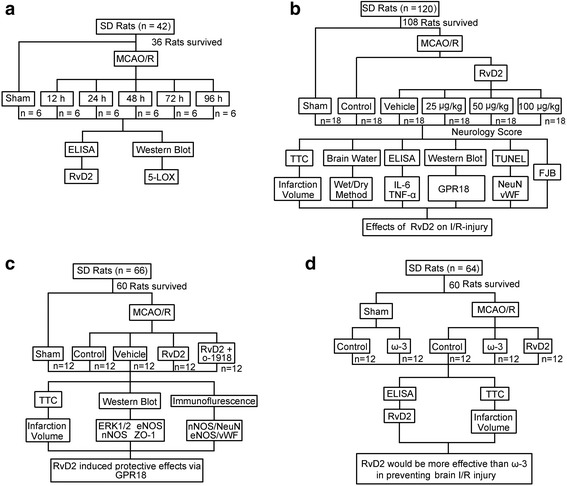
Animal groups. **a** The first experiment was designed to show the time course of the protein level of 5-LOX, phosphorylation of 5-LOX, and levels of endogenous RvD2 after MCAO/R and identify the suitable time point for the second experiment. **b** The second experiment was designed to observe the effects of intervention with exogenous RvD2 at different doses on I/R brain injury after MCAO/R in SD rats. **c** The third experiment was designed to identify the potential mechanisms of the alleviation of I/R-brain injury by RvD2. **d** The fourth experiment was designed to compare the effects of RvD2 on I/R with those of ω3-polyunsaturated fatty acids in vivo

In experiment 1, 36 adult male SD rats (42 rats were used, but only 36 rats survived after the MCAO modeling surgery) were randomly assigned into 6 groups, the sham group and the experimental groups arranged by time: 12, 24, 48, 72, and 96 h (*n* = 6 per group). Under an operating microscope, the focal cerebral ischemia was accomplished by right-sided endovascular middle cerebral artery occlusion (MCAO). Briefly, the rats were anesthetized, and the rectal temperature was maintained at 37 ± 0.5 °C with a heating lamp. Then, the right common carotid arteries, external carotid arteries, and internal carotid arteries were revealed surgically. Next, a piece of 4-0-monofilament nylon suture with a blunted tip coated with polylysine was used to occlude the middle cerebral artery (MCA). The nylon suture was inserted through the right common carotid arteries and from the external carotid arteries into the internal carotid arteries until the tip occluded the proximal stem, indicating MCAO induction. After ischemia for 2 h, the filament was withdrawn to allow reperfusion. At 12, 24, 48, 72, and 96 h of ischemia after reperfusion, brain tissues were obtained for analysis (Fig. 8a).

In experiment 2, 108 adult male SD rats (120 rats were used, 108 rats survived after the MCAO modeling surgery) were randomly divided into six groups: sham group, MCAO/R group, MCAO/R + vehicle group, MCAO/R + RvD2 (25 μg/kg) group, MCAO/R + RvD2 (50 μg/kg) group, and MCAO/R + RvD2 (100 μg/kg) group (*n* = 18 per group). After the indicated treatments, rats were euthanized, and the brain tissues were separated and taken for analysis (Fig. 8b).

In experiment 3, 60 adult male SD rats (66 rats were used, but only 60 rats survived after the MCAO modeling surgery) were randomly assigned into five groups: sham group, MCAO/R group, MCAO/R + vehicle group, MCAO/R + RvD2 (50 μg/kg) group, and MCAO/R + RvD2 (50 μg/kg) + O-1918 group (*n* = 12 per group). After the indicated treatments, rats were euthanized, and the brain tissues were separated and taken for analysis (Fig. 8c).

In experiment 4, 60 adult male SD rats (64 rats were used, 60 rats survived after the surgery) were divided into five groups: sham group, sham + ω-3 group, MCAO/R group, MCAO/R + ω-3 group, and MCAO/R + RvD2 (50 μg/kg) group (n = 12 per group). After the indicated treatments, rats were euthanized, and the brain tissues were separated and taken for analysis (Fig. 8d).

### Ischemic penumbra dissection

After the indicated treatments, the rats were transcardially perfused with ice-cold PBS. Then the brain was quickly removed and frozen in stainless steel brain matrices (− 20 °C) for 10 min, and the cerebellum and olfactory bulb were discarded. As described previously [33], the ischemic penumbra was dissected. In brief, the brain was sliced into three slices from the anterior tip of the frontal lobe. Both the front and back slices were sliced at 3 mm thickness, while the middle slice was sliced at 4 mm thickness. The middle slice was then cut in the ischemic hemisphere 2 mm from the midline longitudinally. To separate the core from the penumbra, a transverse diagonal cut was made at the 2 o’clock position. Finally, the cerebral cortical penumbra was harvested for Western blot and immunohistochemistry analyses.

### Extraction and culture of neurons

Primary neurons were prepared from the brains of fetal rats (from 16 to 18-day pregnant SD rats). Every effort we made was to reduce the number of embryos used and their suffering. First, we removed the meninges and blood vessels. Next, the brains were digested with 0.25% trypsin (with ethylenediaminetetraacetic acid [EDTA]) for 5 min and washed with PBS 3 times to terminate the digestion. Then, we suspended the tissue in PBS, filtered the cell suspension, and centrifuged the suspension at 500×g for 5 min. The neurons were then suspended and plated on a poly-L-lysine-coated culture vessel and cultured in Neurobasal-A medium containing 2% B27, 2 mM L-glutamine, 50 U/ml penicillin, and 50 U/ml streptomycin (all from Gibco, Carlsbad, CA, USA). Half of the medium was renewed every 2 days for 2 weeks. Finally, the neurons were exposed to the indicated treatments and harvested.

### Extraction and culture of microglia

Primary microglia were prepared from brains of 1-day-old newborn rats. The digestion process was similar to that of neurons. After centrifugation, cell deposits were suspended in DMEM/F12 containing 10% fetal bovine serum, 1 mM sodium pyruvate, 2 mM L-glutamine, 100 mM nonessential amino acids, 50 U/ml penicillin, and 50 U/ml streptomycin (all from Gibco, Carlsbad, CA, USA). Then cells were seeded into a 150 cm^2^ culture flask in the fresh DMEM/F12 medium. Half of the medium was renewed every 2 days. Two weeks after initial seeding, a confluent polylayer of glial cells could be observed, and microglia could be separated from astrocytes by shaking the flask for 4 h at 150 rpm. The cell suspension was collected and centrifuged. Finally, the microglia were suspended and plated on poly-L-lysine-coated 12-well plates in the fresh medium.

### BMVEC culture

The human cerebral microvascular endothelial cell line hCMEC/D3 was purchased from the Cell Bank of the Chinese Academy of Sciences (Shanghai, China). hCMEC/D3 cells were cultured as routine in 1640 medium (Hyclone, SH30809.01B) supplemented with 10% heat-inactivated fetal bovine serum.

### Astrocyte culture

The human astrocyte cell line was obtained from ScienCell Research Laboratories (Cat #1800). According to the manufacturer’s instructions, astrocytes were plated on a poly-L-lysine-coated culture vessel and cultured in astrocyte medium (ScienCell, Cat #1801).

### Antibodies

Rabbit anti-GPR18 polyclonal antibody (SAB4501252) was provided by Sigma. Rabbit anti-albumin polyclonal antibody (ab51422), rabbit anti-eNOS polyclonal antibody (ab66127), rabbit anti-nNOS monoclonal antibody (ab76067), rabbit anti-5-LOX monoclonal antibody (ab169755), mouse anti-NeuN antibody (ab104224), mouse anti-GFAP antibody (ab10062), and mouse anti-von Willebrand factor (vWF) antibody (ab68545) were purchased from Abcam (Cambridge, MA, USA). Rabbit anti-5-LOX (phospho-Ser663) polyclonal antibody (P12527) was purchased from Biorbyt (Cambridge, CA, UK). Rabbit anti-ERK1/2 (phospho-Thr202/Tyr204) antibody (#4370) and monoclonal rabbit anti-β-tubulin antibody (#2146) were provided by Cell Signaling Technology (Danvers, MA, USA). Rabbit anti-ZO-1 polyclonal antibody (RA231621) was purchased from Thermo Fisher (Waltham, MA, USA). Goat anti-rabbit IgG HRP (sc-2004) was purchased from Santa Cruz Biotechnology (CA, USA). Alexa Fluor-488 donkey anti-rabbit IgG antibody (A21206) and Alexa Fluor-555 donkey anti-mouse IgG antibody (A31570) were purchased from Invitrogen (Carlsbad, CA, USA).

### Reagents

RvD2 (item no. 10007279) was purchased from Cayman (Ann Arbor, MI, USA). Fish oil (cholesterol free) containing 30% ω-3 fatty acids was obtained from Nature’s Bounty, Inc.; 1000 mg fish oil contained 300 mg ω-3 fatty acids, including 180 mg EPA and 120 mg DHA. O-1918 (ab120405) was purchased from Abcam. All of the reagents for SDS-polyacrylamide gel electrophoresis and Western blot were purchased from Bio-Rad (Richmond, CA, USA). Phenylmethylsulfonyl fluoride (PMSF), aprotinin, Triton X-100, dithiothreitol (DTT), glycerol, Tween 20, BSA (fraction V), and TTC (T8877) were purchased from Sigma Chemical Co. (St. Louis, MO, USA). Chemiluminescence protein labeling reagents in immunoblots were purchased from Thermo Fisher. The terminal deoxynucleotidyl transferase dUTP nick end labeling (TUNEL) staining kit (DeadEnd fluorometric kit) was purchased from Promega (Madison, WI, USA). Fluoro-Jade B (FJB) was purchased from Histo-Chem, Inc. (Jefferson, AR, USA), and 4′,6-diamidino-2-phenylindole (DAPI) was obtained from SouthernBiotech (Birmingham, AL, USA).

### ELISA

The brain concentrations of RvD2, IL-6, and TNF-α were determined by an RvD2 ELISA kit (Cayman, 501,120), rat IL-6 kit (USCN, Ra20607), and rat TNF-α kit (USCN, Ra20035). These assays were performed according to the manufacturers’ instructions.

### Neurobehavioral scores

At 72 h after MCAO/R, the behavioral impairment was evaluated utilizing a previously published scoring system, and the appetite, activity, and neurological defects of all rats were monitored in experiment 2 [34] (Table 1). All behavioral tests were performed by investigators who were blinded to the group assignment.

**Table 1:** Behavior and activity scores

Category	Behavior	Score
Appetite	Finished meal	0
	Left meal unfinished	1
	Scarcely ate	2
Activity	Walk and reach at least three corners of the cage	0
	Walk with some stimulations	1
	Almost always lying down	2
Deficits	No deficits	0
	Unstable walk	1
	Impossible to walk	2

### Brain edema

As described previously [35], the wet/dry method was used to evaluated the brain edema. Briefly, the brain tissues were collected, and the samples of brainstem and cerebellum were weighed immediately as the wet weight. The samples were kept at 100 °C for 72 h and then weighed again to obtain the dry weight. The percentage of water content was calculated as follows: [(wet weight − dry weight)/wet weight] × 100%.

### TUNEL and FJB staining

First, all the paraffin sections (4–6 μm) for detection were dried for 1 h at 70 °C and deparaffinized in xylene and graded ethanol solutions (100%, 95%, 90%, 80%, and 70%).

According to the manufacturer’s protocol, TUNEL staining was used to detect the cell apoptosis in brain tissue. Then, the TUNEL staining slides were incubated with von Willebrand factor (vWF) and NeuN antibody at 4 °C overnight, followed by the incubation of the indicated secondary antibody at 37 °C for 1 h. Finally, the slides were visualized by a fluorescence microscope (Olympus BX50/BX-FLA/DP70), and the TUNEL-positive cell count was obtained, which was defined as the average number of TUNEL-positive cells in each section counted in six microscopic fields.

FJB staining was used as a marker of neuronal injury and performed as follows. After deparaffinizing and rehydrating of the brain sections, the slides were incubated with 80% alcohol containing 1% NaOH for 1 min, followed by incubation in 0.06% potassium permanganate for 15 min. Then, the slides were rinsed in deionized water and immersed in 0.001% FJB working solution (0.1% acetic acid) for 30 min at room temperature, after which they were washed and dried in an incubator (50–60 °C) for 10 min. Then, the sections were dehydrated with xylene and covered by neutral balsam (Distyrene Plasticiser Xylene, Sigma-Aldrich, Inc., St. Louis, MO). The stained tissue sections were visualized by a fluorescence microscope, and the numbers of FJB positive cells per mm^2^ were counted in the selected fields.

### Western blot analysis

Western blot analysis was conducted as described previously [36]. The brain samples were mechanically lysed in precooling RIPA lysis buffer (Beyotime) for 30 min and centrifuged at 12,000 rpm for 20 min. After the supernatant was collected, the protein concentrations were determined by the bicinchoninic acid (BCA) protein assay kit (Beyotime). Protein samples (50 μg) were separated by a 12% SDS-polyacrylamide gel and transferred to a polyvinylidene difluoride (PVDF) membrane (Millipore). The membrane was blocked by 5% nonfat milk for 1 h at room temperature, incubated overnight at 4 °C with primary antibodies against GPR18 (1:1000), nNOS (1:2000), eNOS (1:2000), ZO-1 (1:2000), Phos-ERK1/2 (1:1000), 5-LOX (1:2000), Phos-5-LOX (1:500), and β-Tubulin (1:5000), and then incubated with the indicated horseradish peroxidase (HRP)-conjugated secondary antibodies (Santa Cruz, 1:5000) for 2 h at room temperature. The band signal was detected using SuperSignal West Pico chemiluminescent substrate (Thermo Fisher Scientific). The protein quantification was analyzed by ImageJ software and normalized to that of the loading control. The phosphorylated level of proteins was normalized to its total protein level.

### Immunofluorescence staining

The protein levels of nNOS and eNOS in neurons and BMVECs were detected by immunofluorescence staining. After incubation with the primary antibodies for nNOS, eNOS, NeuN, and vWF overnight at 4 °C, the indicated secondary antibodies (Alexa Fluor 488 donkey anti-rabbit IgG antibody and Alexa Fluor 555 donkey anti-mouse IgG antibody) were incubated for 1 h at 37 °C, followed by washing with PBST three times. Then, the sections were protected by cover slips with anti-fading mounting medium containing DAPI. Normal rabbit/mouse IgG served as the negative control for the immunofluorescence assay (data not shown). Finally, sections were observed by a fluorescence microscope (Olympus BX50/BX-FLA/DP70), and the relative fluorescence intensity was analyzed by ImageJ software.

### TTC staining

After MCAO/R for 72 h, the rats were sacrificed under anesthetization. The brains were removed and frozen quickly in precooling stainless-steel brain matrices (− 20 °C) for 10 min. Next, the brains were sliced coronally at 2 mm thickness for 5 slides starting from the frontal pole, and the cerebellum and olfactory bulb sections were discarded. Slices were then immersed in 2.0% TTC solution for 30 min at 37 °C, followed by washing three times with saline. Then the sections were fixed in 10% formalin and captured with a digital camera. Finally, we calculated the infarct volume as described previously [37]. In brief, the mean of the total infarction of each section was calculated as the average of the area on the rostral and the caudal side. Total infract volumes were calculated by adding the average area of each section and then multiplying by 2 mm (thickness of the sections). Infarct volume was also calculated as a percentage of the ipsilateral hemispheric volume by an investigator who was blinded to the group assignment.

### Drug administration

The ω-3 fatty acid pretreatment rats received an oral gavage of 30% ω-3 fatty acids at 1 g/kg once every 24 h for the indicated time. RvD2 and O-1918 were dissolved in PBS and DMSO, respectively, at the stock concentration and diluted to 50 μg/kg and 3 μmol/kg by PBS per the manual, followed by intraperitoneal injection after the MCAO/R model was established for 72 h. The RvD2 dosage was based on the research on RvD1 [38] and confirmed by experiment 1 (Fig. 1). In the in vitro experiment, RvD2 was used to treat cultured neurons, microglia, astrocytes, and BMVECs immediately after OGD/R at 10 μmol/L.

### Statistical analyses

The frequency distribution for the neurobehavioral score assay was determined, and the neurobehavioral score was shown as the median, with an interquartile range. All other data were presented as mean ± SEM and analyzed by one-way ANOVA followed by Dunnett’s test. GraphPad Prism 7.00 was used for all statistical analysis. *P* < 0.05 was considered to be a significant difference.

## Abbreviations

BBB: Blood-brain barrier
BMVEC: Brain microvascular endothelial cell
CNS: Central nervous system
CSF: Cerebrospinal fluid
EBI: Early brain injury
ELISA: Enzyme-linked immunosorbent assay
eNOS: Endothelial NOS
GPCR: G protein-coupled receptor
I/R: Ischemia/reperfusion
IL-6: Interleukin 6
LOX: Lipoxygense
MCAO: Middle cerebral artery occlusion
MCAO/R: Middle cerebral artery occlusion and reperfusion
nNOS: Neuronal NOS
ROS: Reactive oxygen species
RvD2: Resolvin D2
SAH: Subarachnoid hemorrhage
TNF-α: Tumor necrosis factor α
ZO-1: Zonula occludens 1
